# Multiple Primary Melanomas: Clinical and Genetic Insights for Risk-Stratified Surveillance in a Tertiary Center

**DOI:** 10.3390/jpm15080343

**Published:** 2025-08-01

**Authors:** Marta Cebolla-Verdugo, Francisco Manuel Almazán-Fernández, Francisco Ramos-Pleguezuelos, Ricardo Ruiz-Villaverde

**Affiliations:** 1 Department of Dermatology, Hospital Universitario San Cecilio, 18016 Granada, Spain; marta.cebolla.sspa@juntadeandalucia.es (M.C.-V.); franciscom.almazan.sspa@juntadeandalucia.es (F.M.A.-F.); 2 Instituto de Investigación Biosanitaria de Granada (ibs.GRANADA), 18012 Granada, Spain; 3 Department of Pathology, Hospital Universitario San Cecilio, 18016 Granada, Spain; franciscom.ramos.sspa@juntadeandalucia.es

**Keywords:** multiple primary melanomas, personalized medicine, melanoma surveillance, genetic risk, stratified follow-up

## Abstract

**Background:** Patients diagnosed with melanoma are at increased risk of developing multiple primary melanomas (MPMs). Identifying clinical and genetic factors associated with MPM is critical for implementing personalized surveillance strategies. This study aims to describe the clinical, histopathological, and genetic characteristics of patients with MPM managed in a tertiary hospital and to contextualize findings within the current literature. **Methods**: We conducted a retrospective review of patients diagnosed with two or more primary melanomas between 2010 and 2023 at a tertiary dermatology unit. Demographic data, personal and family cancer history, phototype, melanoma characteristics, genetic testing, staging, treatments, and outcomes were collected. These data were compared with findings from the recent literature. **Results**: Thirteen patients (ten males, three females; median age: 59 years) were found to have a total of 33 melanomas. Most patients had Fitzpatrick phototype II and no immunosuppression. The number of melanomas per patient ranged from two to five. Synchronous lesions were observed in two patients. Common locations included the trunk and extremities. Histologically, 57% were in situ melanomas, and subsequent melanomas were generally thinner than the index lesion. Two patients showed progression to advanced disease. One patient was positive for MC1R mutation; the rest were negative or inconclusive. Additional phenotypic and environmental risk factors were extracted from patient records and are summarized as follows: Ten patients (76.9%) had Fitzpatrick skin phototype II, and three (23.1%) had phototype III. Chronic occupational sun exposure was reported in four patients (30.8%), while five (38.5%) recalled having suffered multiple sunburns during childhood or adolescence. Eight patients (61.5%) presented with a total nevus count exceeding 50, and five (38.5%) exhibited clinically atypical nevi. None of the patients reported use of tanning beds. **Conclusions**: Our findings are consistent with the existing literature indicating that patients with MPM often present with thinner subsequent melanomas and require long-term dermatologic follow-up. The inclusion of genetic testing and phenotypic risk factors enables stratified surveillance and supports the application of personalized medicine in melanoma management.

## 1. Introduction

Melanoma is one of the most aggressive forms of skin cancer, with a rising global incidence. Although survival rates have improved in recent decades due to prevention campaigns, health education, and therapeutic advances, longer survival increases the probability of developing a second primary melanoma. The reported incidence of multiple primary melanomas (MPMs) varies from 1.1% to 20.4% across studies, with the highest incidence in Australia and the lowest in Sweden [1]. In the United States, the annual increase was 2.7% between 1986 and 2007 [1]. In Europe, the incidence is approximately 25 per 100,000 inhabitants, compared to 60 per 100,000 in Australia [1].

The development of MPM is influenced by both environmental and genetic factors. Established risk factors include a personal or family history of melanoma, dysplastic nevi, Fitzpatrick skin types I and II, excessive ultraviolet radiation exposure, use of tanning beds, male sex, and older age [2]. Mutations in the CDKN2A and CDK4 genes are detected in up to 20% of familial MPM cases [3].

In Spain, the Granada Cancer Registry contributes to national surveillance efforts and offers important epidemiological insights. In a recent analysis, 2446 cases of cutaneous melanoma were diagnosed between 1985 and 2017 in patients aged 15 years or older. A significant increase in incidence was observed in both men and women (APC: 4.4% and 3.7%, respectively), predominantly in superficial spreading and nodular melanomas, and particularly among those with a Breslow index < 1 mm. Mortality rates also increased, especially in men and individuals aged ≥65 years [4].

These findings are consistent with national trends and emphasize the critical need for early detection strategies. The same registry study revealed notable differences in primary tumor localization by sex and age group. In men, the most common sites were the trunk and head–neck region, whereas in women, the lower extremities were most frequently affected. Survival rates have improved for localized disease, but advanced-stage melanomas still show poor prognoses. Five-year net survival for localized melanoma reached 95.6%, while survival fell sharply to 65.1% for regional disease and 20.7% for distant metastases [4]. These survival disparities reinforce the importance of early diagnosis and structured follow-up to identify secondary melanomas during earlier stages. Notably, diagnostic delay and tumor ulceration were among the factors most strongly associated with poor outcomes. Moreover, comorbidities and socioeconomic variables have been linked to delayed presentation and decreased survival, further supporting the need for tailored approaches in high-risk groups [4].

MPMs are classified as synchronous when diagnosed within the first three months after the initial melanoma, or metachronous when detected thereafter. The proportion of synchronous melanomas ranges from 5% to 66% of reported cases [1]. The trunk is the most frequent site for the first and second melanomas, with superficial spreading melanoma being the most common histological variant in both cases. Typically, the Breslow thickness of the second melanoma is lower, suggesting earlier detection in patients under follow-up [5]. Most MPMs occur within the first five years after the initial diagnosis, highlighting the necessity for long-term surveillance protocols [6]. Genetic mutations (e.g., CDKN2A), high nevus density, atypical nevi, older age, male sex, fair skin, and inadequate sun protection are key contributing factors [7].

This study presents a retrospective analysis of MPM patients treated in a tertiary dermatology unit, aiming to describe clinical, pathological, and genetic features and propose a risk-adapted follow-up model aligned with the principles of personalized medicine.

## 2. Materials and Methods

We conducted a retrospective, observational study at the Dermatology Department of Hospital Universitario San Cecilio (Granada, Spain), including patients diagnosed with multiple primary melanomas (MPMs) between January 2010 and December 2023. MPM was defined as histologically confirmed, distinct melanomas arising independently in a single patient. Lesions were classified as synchronous if diagnosed within three months and metachronous if diagnosed later.

All eligible cases were identified through the institutional melanoma registry, curated by the Dermatopathology Service. This registry compiles demographic, clinical, and histopathological data systematically. Exclusion criteria included recurrent tumors, incomplete data, re-excision specimens, sentinel lymph node biopsies, and punch biopsies not intended for definitive diagnosis. Only invasive or in situ melanomas with histopathological confirmation were included.

Demographic and clinical variables extracted included age at diagnosis, sex, Fitzpatrick phototype, personal/family history of melanoma or other cancers, immunosuppression status, total number of melanomas, anatomical distribution, histologic subtype, Breslow thickness, ulceration, mitotic index, and AJCC staging.

Genetic testing was offered to patients who met institutional criteria based on national guidelines for hereditary melanoma. Specifically, testing was performed in individuals with either (a) two or more primary melanomas, or (b) a family history including two or more first- or second-degree relatives with melanoma and/or pancreatic cancer or astrocytoma. All patients underwent genetic counseling before and after testing through the institution’s hereditary cancer risk program. The germline gene panel included high- and moderate-penetrance genes associated with familial melanoma: *CDKN2A*, *CDK4*, *BAP1*, *POT1*, *BRCA2*, *TERT*, and *MC1R*.

## 3. Results

During the study period, a total of 442 biopsy samples were recorded, of which 387 met the inclusion criteria. These corresponded to 352 unique patients, among whom 13 were diagnosed with more than one primary melanoma, representing 3.7% of patients with multiple primary melanomas in our cohort.

We identified 13 patients with multiple primary melanomas diagnosed between 2010 and 2023, comprising 10 men (77%) and 3 women (23%), with a median age of 59 years (range: 40–83). The majority had Fitzpatrick phototype II, with one patient reporting a prior diagnosis of breast cancer. No patient in our cohort was immunosuppressed at the time of melanoma diagnosis. Detailed clinical and pathological characteristics are summarized in Table 1.

A total of 33 melanomas were diagnosed in these patients, with a median of 2.5 melanomas per individual (range: 2–5). Eleven patients (84.6%) had metachronous melanomas, while two cases (15.4%) presented synchronous tumors. The interval between melanoma diagnoses ranged from 3 months to 8 years. Anatomic distribution favored the trunk (45%) and extremities (40%), consistent with known UV exposure patterns and predilection sites in fair-skinned populations. Two patients had a family history of melanoma. No patients had a family history of pancreatic cancer. No patients had immunosuppression at baseline.

Additional phenotypic and environmental risk factors were extracted from patient records and are summarized as follows: Ten patients (76.9%) had Fitzpatrick skin phototype II, and three (23.1%) had phototype III. Chronic occupational sun exposure was reported in four patients (30.8%), while five (38.5%) recalled having suffered multiple sunburns during childhood or adolescence. Eight patients (61.5%) presented with a total nevus count exceeding 50, and five (38.5%) exhibited clinically atypical nevi. None of the patients reported use of tanning beds.

Histologically, superficial spreading melanoma was the most prevalent subtype, followed by lentigo maligna and nodular melanomas. In situ melanomas constituted 57% of all lesions, reflecting a high detection rate of early-stage tumors likely facilitated by regular surveillance. Subsequent melanomas exhibited consistently lower Breslow thickness compared to index melanomas. None of the patients presented ulceration or a high mitotic index in more than one lesion. Lymphovascular invasion was not observed.

Staging at diagnosis revealed early-stage tumors in the majority of patients. Two patients progressed to advanced disease: one to stage IIIB with nodal involvement, and another to stage IIIC with locoregional cutaneous metastases. These cases highlight the heterogeneity of clinical evolution in MPM and the potential for aggressive behavior even with vigilant follow-up.

Genetic testing was performed in five patients, selected according to institutional criteria. Only one patient tested positive for an MC1R variant, while others showed negative or inconclusive results. MC1R was included in the genetic panel due to its association with low-to-moderate penetrance risk for melanoma, particularly in individuals with a red hair phenotype, fair skin, and increased UV sensitivity. Although not a high-penetrance gene like CDKN2A, its inclusion is supported by growing evidence in the context of risk stratification.

## 4. Discussion

This case series presents a cohort of patients with multiple primary melanomas (MPMs) managed at our tertiary care institution, providing a detailed clinical, histological, and genetic characterization. Our findings align with the existing literature while highlighting local epidemiological trends and novel opportunities for personalized management.

MPM remains a relatively uncommon but clinically significant presentation of cutaneous melanoma. In our series, patients presented with two to five distinct melanomas over time, predominantly on the trunk and extremities. This pattern reflects previous reports indicating a higher likelihood of subsequent melanomas in sun-exposed areas in individuals with high nevus density and lighter skin phototypes [1,2]. The high frequency of the superficial spreading subtype and the prevalence of in situ lesions among subsequent melanomas support the role of intensified dermatologic surveillance following initial diagnosis. This finding is consistent with the notion that early detection leads to the identification of thinner and earlier-stage melanomas in high-risk patients [3].

Our series also reinforces the distinction between synchronous and metachronous MPM. The predominance of metachronous lesions (diagnosed more than three months apart) in our cohort is in line with the broader literature, which suggests that approximately 80–90% of MPMs follow a metachronous pattern [1,8,9]. The time interval between diagnoses underlines the need for sustained long-term follow-up, particularly in patients with known phenotypic and genetic risk factors. Current guidelines emphasize five-year monitoring, yet some studies advocate for lifelong surveillance given the ongoing risk of new primary lesions well beyond that window [6,10].

The inclusion of genetic testing in our study, though limited to a subset of patients, is increasingly relevant in the context of precision oncology. Only one patient tested positive for an MC1R variant, while others showed negative or inconclusive results. Although we did not detect pathogenic CDKN2A or CDK4 mutations, the literature indicates their presence in up to 20% of familial MPM cases [3]. Other high- and moderate-penetrance genes—such as BAP1, POT1, BRCA2, and TERT—were also included, reflecting an expanded institutional approach to hereditary melanoma risk assessment. Based on current evidence, appropriate patient selection is critical when offering genetic testing. Environmental factors—such as cumulative UV exposure and sunburn history—may play a predominant role in many patients with MPM. Future research aimed at better characterizing familial melanoma, candidate genes, and gene–environment interactions will be essential to advance our understanding of melanoma susceptibility.

Recent insights into the molecular biology of melanoma suggest that certain MPM subtypes—particularly those associated with lentigo maligna or desmoplastic variants—may exhibit a high mutational burden and unique gene expression profiles, including frequent alterations in NF1 and TP53 [4,11,12,13]. These findings have therapeutic implications, particularly regarding responsiveness to immune checkpoint inhibitors. Although systemic therapy was not required in most of our cases, understanding the molecular landscape of MPM could inform decisions about adjuvant therapy and identify candidates for clinical trials.

Our findings are in line with those of Carugno et al. [14], who examined 333 patients with multiple and/or familial melanomas. They reported a higher prevalence of MPMs in patients over 60 years, with a greater nevus burden observed in younger individuals. Moreover, 66.9% of second melanomas were detected by dermatologists, underscoring the critical role of structured surveillance. Pathogenic or likely-pathogenic variants were identified in 4.3% of cases, primarily in CDKN2A. A progressive decrease in Breslow thickness was noted in subsequent melanomas, mirroring the trend observed in our cohort.

At our institution, melanoma follow-up is tailored to the AJCC stage. High-risk patients undergo digital dermoscopy (Fotofinder) every 6 months, coinciding with routine dermatologic–oncologic visits. In situ and stage IA melanomas are followed with clinical exams every 6–12 months for 3–10 years. Intermediate and high-stage cases (IB–III) receive imaging and laboratory evaluations at 3–6-month intervals initially, followed by annual assessments. Patients with familial melanoma or dysplastic nevus syndrome are monitored semiannually with total-body photography and dermoscopy.

Importantly, our study reinforces the importance of comprehensive patient education and adherence to surveillance programs. The psychological burden of recurrent melanoma diagnoses, fear of progression, and the complexity of managing multiple surgical sites demand a multidisciplinary approach. This includes not only dermatologists and oncologists but also geneticists, psychologists, and specialized nursing teams. Strategies such as total-body photography, digital dermoscopy, and artificial intelligence-assisted image analysis are emerging tools that could enhance follow-up efficiency and lesion detection [15].

Limitations of our study include the retrospective design, relatively small sample size, and limited availability of genetic data. However, the strength lies in the detailed longitudinal follow-up of a real-world cohort within a high-volume tertiary care center. Future directions include prospective studies integrating clinical, dermoscopic, histological, and genomic data to refine risk prediction models.

In summary, MPM presents unique diagnostic, therapeutic, and surveillance challenges. Our study supports a model of stratified follow-up, tailored to individual phenotypic and genotypic profiles. Advances in molecular diagnostics, combined with structured long-term surveillance, offer the opportunity to optimize outcomes and quality of life in this vulnerable patient population.

## 5. Conclusions

Multiple primary melanomas represent a distinct clinical and biological entity with specific risk factors and progression patterns. This study reinforces the need for comprehensive, individualized approaches in surveillance and management. Integrating phenotypic characteristics with genetic profiling allows the development of personalized follow-up strategies. Early detection of subsequent melanomas, facilitated by intensified surveillance, significantly impacts prognosis. Long-term monitoring and interdisciplinary coordination are essential to optimize outcomes in this high-risk population.

## Figures and Tables

**Table 1:** Summary of clinical and pathological characteristics of patients with multiple primary melanomas (MPMs) diagnosed and managed at a tertiary hospital from 2010 to 2023. The table includes data on demographics, family and personal history of cancer, Fitzpatrick skin phototype, total number and anatomical distribution of melanomas, temporal sequence of diagnoses, results of genetic testing, AJCC staging, use of adjuvant therapies, and evidence of disease progression. Synchronous melanomas were considered those diagnosed within three months of the previous lesion. Genetic testing was performed in selected patients based on clinical criteria.

Patient No.	Sex	Age (Years)	Chronic Occupational Sun Exposure	Personal History of Melanoma	Multiple Sunburns During Childhood or Adolescence	Skin Phototype	Total Nevus Count Exceeding 50	Total Melanomas
**1**	F	65	Yes	Yes	Yes	II	Yes	2
**2**	M	49	No	No	No	II	No	2
**3**	M	58	No	No	No	II	Yes	2
**4**	F	56	No	No	No	II	No	2
**5**	M	69	Yes	No	Yes	II	Yes	2
**6**	M	83	No	No	No	II	No	2
**7**	M	40	Yes	No	No	III	Yes	3
**8**	M	59	No	No	No	II	No	5
**9**	M	76	No	No	Yes	II	Yes	2
**10**	M	82	Yes	No	Yes	II	Yes	5
**11**	M	67	No	Yes	No	II	No	3
**12**	M	50	No	No	No	III	Yes	2
**13**	F	40	No	No	Yes	II	Yes	4
**Patient No.**	**Anatomic Location(s)**		**Interval Between Melanomas**	**Genetic Findings**	**AJCC Stage(s)**		**Adjuvant Therapy**	**Disease Progression**
**1**	No.1: Right thigh; No.2: Left thigh		6 years	Negative	No.1: IB; No.1: in situ		No	No
**2**	No.1: Right forearm; No.2: Left flank		10 years	Not performed	No.1: in situ; No.2: IA		No	No
**3**	No.1: Back; No.2: Abdomen		10 years	Inconclusive	No.1: IIA; No.2: IA		No	No
**4**	No.1: Right gluteal; No.2: Chest		6 years	Not performed	No.1: IB; No.2: in situ		No	No
**5**	No.1: Left leg; No.2: Lumbar region		1 year	Negative	No.1: IIA; No.2: in situ		No	No
**6**	No.1: Right clavicle; No.2: Posterior cervical		2 months	Not performed	No.1: IIB; No.2: in situ		No	Yes (cutaneous metastasis in 2022 from melanoma No.1: IIB → IIIC)
**7**	No.1: Right scapular; No.2: Posterior cervical; No.3: Abdomen		1 year (both intervals)	MC1R-positive	No.1: IIIC; No.2: IA; No.3: in situ		Yes (No.1), No (Nos.2–3)	No
**8**	No.1: Left lumbar; No.2: Mid back; No.3: Right subscapular; No.4: Central back; No.5: Right flank		Simultaneous (Nos.1–3); 2 years (Nos.4–5)	Not performed	Nos.1–2: in situ; No.3: IA; No.4: in situ; No.5: in situ		No	No
**9**	No.1: Lumbar; No.2: Right scapular		13 years	Not performed	No.1 IB, No. 2 in situ		No	No
**10**	No.1: Left supraclavicular; No.2: Left forearm; No.3: Back; No.4: Right clavicle; No.5: Right shoulder		6 years (Nos.1–2); 7 years (Nos.2–3); 1 month (Nos.3–4); 2 years (Nos.4–5)	Not performed	No.1–3 IA, No.4 IB, No.5 IA		No	No
**11**	No.1: Posterior trunk; No.2: Lateral trunk; No.3: Right gluteal		3 months (Nos.1–2); 4 months (Nos.2–3)	Negative	Nos.1–3: in situ		No	No
**12**	No.1: Left lumbar; No.2: Left flank		3 years	Not performed	No.1: IIIB; No. 2: in situ		Yes (No.1: nivolumab), No (No.2)	Yes (No.1: costal subcutaneous metastasis, 2023)
**13**	No.1: Left forearm; No.2: Right ankle; No.3: Left flank; No.4: Left ankle		7 months (No.1–2); Simultaneous (Nos.2–3); 1 year (Nos.3–4)	Not performed	No.1: IB; Nos.2–4: in situ		No	No

## Data Availability

The raw data supporting the conclusions of this article will be made available by the authors on request.
